# Cd-Resistant Plant Growth-Promoting Rhizobacteria *Bacillus siamensis* R27 Absorbed Cd and Reduced Cd Accumulation in Lettuce (*Lactuca sativa* L.)

**DOI:** 10.3390/microorganisms12112321

**Published:** 2024-11-15

**Authors:** Shaofang Liu, Yushan Huang, Qinyuan Zheng, Mengting Zhan, Zhihong Hu, Hongjie Ji, Du Zhu, Xia Zhao

**Affiliations:** 1 Key Laboratory of Natural Microbial Medicine Research of Jiangxi Province, Jiangxi Science and Technology Normal University, Nanchang 330013, China; liushaofanghnpy@163.com (S.L.); 15874903122@163.com (Y.H.); 18268730345@163.com (Q.Z.); lince_er@163.com (M.Z.); huzhihong426@163.com (Z.H.); 2 State Key Laboratory of Continental Dynamics, Northwest University, Xi’an 710069, China; 3 Key Laboratory of Microbial Resources and Metabolism of Nanchang City, Jiangxi Science and Technology Normal University, Nanchang 330013, China; 4 State Key Laboratory of Nuclear Resources and Environment, East China University of Technology, Nanchang 330013, China; 5 Key Laboratory of Ecological Safety and Sustainable Development in Arid Lands, Chinese Academy of Sciences, Lanzhou 730000, China; zhaoxia@lab.ac.cn

**Keywords:** PGPR, *Bacillus siamensis*, lettuce, Cd-resistant bacteria, bioremediation

## Abstract

The use of plant growth-promoting rhizobacteria (PGPR) for the bioremediation of heavy metal cadmium (Cd) and for enhancing plant growth in Cd-polluted soil is widely recognized as an effective approach. This study aimed to isolate Cd-resistant bacteria with plant growth-promoting (PGP) traits from the rhizosphere of vegetables subjected to metal contamination and to investigate the mechanisms associated with Cd adsorption as well as its impact on Cd uptake in lettuce. Six Cd-resistant bacterial strains were isolated from rhizosphere soil, among which the R27 strain exhibited the highest tolerance to Cd (minimum inhibitory concentration of 2000 mg/L) along with PGP traits, including phosphate solubilization (385.11 mg/L), the production of indole-3-acetic acid (IAA) (35.92 mg/L), and siderophore production (3.34 mg/L). Through a range of physiological, biochemical, and molecular assessments, the R27 strain was classified as *Bacillus siamensis*. This strain demonstrated notable efficiency in removing Cd^2+^ from the growth medium, achieving an efficacy of 80.1%. This removal was facilitated by cell surface adsorption through functional groups such as O–H, C=O, –CO–NH–, and C–O, alongside intracellular Cd accumulation, as evidenced by SEM, TEM, EDX, and FTIR analyses. Pot culture experiments indicated that R27 significantly promoted lettuce seedling growth and helped plants tolerate Cd stress, with the underlying mechanisms likely involving increased antioxidant activities for scavenging reactive oxygen species (ROS) induced by Cd stress, and reduced Cd^2+^ levels in lettuce seedlings to mitigate Cd^2+^ toxicity. These physiological changes were further supported by the down-regulation of genes associated with cadmium transport, including *IRT1*, *Nramp1*, *HMA2*, *HMA4*, *ZIP4*, and *ZIP12*, as well as the significantly reduced root bio-concentration factor (BCF) and translocation factor (TF). In summary, the R27 strain offers considerable potential in the bioremediation of Cd-polluted soils and can serve as a bio-fertilizer to enhance plant growth.

## 1. Introduction

Vegetables offer a wide range of crucial vitamins, minerals, crude fiber, and other important nutrients that are essential for human health, positioning them as a vital food source [1,2]. However, the rapid advancement of industrial activities has intensified the contamination of edible vegetables through urban and industrial sewage, exposing them to heavy metals and associated toxicity [3]. As one of the most commonly consumed vegetables, lettuce (*Lactuca sativa* L.) is extensively cultivated and readily accumulates heavy metals when grown in heavy metal-contaminated farmland [4,5,6,7]. Cd, a non-essential element, is highly toxic to humans and plants, even at low concentrations [8]. When Cd accumulates in the edible portions of vegetables, it can enter the human body through the food chain, ultimately posing a threat to human health [9,10,11].

Cd can inhibit plant growth by affecting various physiological processes. Excessive levels of Cd in plants can reduce photosynthesis by reducing chlorophyll synthesis and accelerating chlorophyll degradation, ultimately leading to a decrease in biomass accumulation [12,13]. Moreover, Cd also triggers ROS, which results in oxidative stress that can denature essential macromolecules [14]. Therefore, it is crucial to establish effective methods to limit the excessive absorption and accumulation of Cd in vegetables, thus ensuring the safety of vegetables grown in Cd-contaminated soils.

Recently, there has been a significant increase in attention toward the investigation and application of plant growth-promoting rhizobacteria (PGPR) in bioremediation strategies [15]. PGPR refers to a category of bacteria that colonize the plant rhizosphere and promote plant growth by synthesizing enzymes like 1-aminocycloproane-1-carboxylic acid (ACC) deaminase, phytohormones like indole-3-acetic acid (IAA), and by promoting nutrient absorption by means of phosphate solubilization, which also plays important roles in enhancing plant tolerance to multiple environmental stresses, including heavy metal stress [16,17,18,19]. Certain Cd-resistant PGPR possess the ability to enhance plant growth while also decreasing the accumulation of Cd in plants. For instance, the strains *Flavisolibacter* sp. 77 and *Bacillus* sp. T36 were able to reduce Cd content in the shoot tissues of tomato plants by 33% and 23%, respectively, and in the root tissues by 24% and 17% [20]. Similarly, *Enterobacter* sp. Zm-123 showed the capacity to decrease Cd content in the edible parts of rapeseed by 36%, while also increasing the biomass of those plants by 20% [21]. The plant growth-promoting *Bacillus* sp. strain SDA-4 was demonstrated to enhance Cd tolerance in lettuce (*Spinacia oleracea* L.) by increasing its biomass and reducing Cd uptake in the plants [22].

Lettuce has been identified as a suitable plant for investigating the enrichment traits of plants under Cd stress [23], so lettuce was selected in order to study the interaction of plants and PGPR under Cd stress. This research focused on the isolation and screening of Cd-resistant PGPR, extracted from the rhizosphere soil of vegetables located near a uranium mining site. The selected strains were characterized based on physiological and biochemical assessments, as well as 16S rDNA gene sequence analysis. Additionally, scanning electron microscopy (SEM), transmission electron microscopy (TEM), energy-dispersive spectroscopy (EDX), and Fourier transform infrared spectroscopy (FTIR) were employed to investigate the mechanisms of Cd^2+^ adsorption by the Cd-resistant strains in a liquid medium. Furthermore, the effects of the Cd-resistant strains on the growth of lettuce seedlings and accumulation of Cd^2+^ by these plants were evaluated. This study not only presents new strains as resources for the remediation of Cd-contaminated soil but also establishes a theoretical foundation for the application of PGPR in reducing Cd^2+^ absorption in vegetables, thereby contributing to the sustainable production of lettuce.

## 2. Materials and Methods

### 2.1. Rhizosphere Samples and Isolation of Cd-Resistant Bacteria

Samples from the rhizosphere soil of vegetable fields adjacent to a uranium mine in Fuzhou, Jiangxi Province (116°10.42″ E, 27°32.53″ N), were collected. Cd-resistant bacteria were isolated using the dilution plate method [22]. A 5 g sample of soil was mixed with 45 mL of sterile water and shaken for 1 h. Subsequently, 5 mL of the supernatant was transferred to a beef extract–peptone liquid medium (LB) containing 1000 mg/L Cd^2+^, which was then incubated at 30 °C with shaking at 150 rpm for 48 h, after which it was serially diluted. 100 μL from the 10^−3^ and 10^−6^ dilutions were spread on LB solid plates containing 500 mg/L CdCl_2_ to isolate bacterial colonies. Strains exhibiting distinct morphologies were selected and purified through repeated streaking.

### 2.2. Characterization of Cd Tolerance

To screen for highly Cd-tolerant strains among the purified bacterial isolates, the minimum inhibitory concentration (MIC) of CdCl_2_ was determined, following the approach of Shahid et al. [22]. Each purified isolate was cultivated in an LB liquid medium, and the cell density was standardized by determining growth using a spectrophotometer at 600 nm. Subsequently, 100 μL of each isolate was spread on LB solid plates containing varying concentrations of CdCl_2_ (0, 500, 1000, 1500, 2000, and 2500 mg/L). The MIC was determined as the lowest concentration of Cd at which the isolates completely failed to grow colonies on the LB plate containing Cd. All the experiments were repeated three times.

### 2.3. Selection of Cd-Resistant PGPR

Further screening was conducted through a qualitative analysis of PGP traits, specifically focusing on phosphate solubilization, siderophore production, and IAA synthesis. The measurement of phosphate solubilization and siderophore production adhered to the procedures established by Nautiyal [24] and Schwyn and Neilands [25]. IAA production levels by each isolate were quantitatively assessed using the Salkowski reagent, as described by Glickmann and Dessaux [26]. All the experiments were performed in triplicate.

### 2.4. Bacterial Identification

The R27 strain was selected for further examination due to its notable resistance to Cd and its plant growth-promoting (PGP) characteristics. The physiological and biochemical characteristics of the R27 strain were assessed to facilitate its identification. This investigation included a series of experiments, such as the Gram stain test, starch hydrolysis, gelatin hydrolysis, indole reaction, methyl red test, oxidase test, hydrogen peroxide reaction, Voges–Proskauer (V-P) test, nitrate utilization assessment, and H_2_S production.

Genomic DNA was extracted from the R27 strain using a Bacterial Genomic DNA Extraction Kit (Solarbio^®^, Beijing, China) following the manufacturer’s instructions. Molecular identification was conducted by amplifying the 16S rRNA gene with universal primers 27F (5′-AGAGTTTATCCTGGGCTCAG-3′) and 1492R (5′-GGTTACCTTGTTACGACTT-3′), as described in [27]. The PCR reaction mixture, with a total volume of 50 µL, comprised 5 μL of 10 × Ex Taq Buffer, 2 μL of Ex Taq polymerase, 2.5 μL of 27F, 2.5 μL of 1492R, 2 μL of DNA template, and 36 μL of ddH_2_O. The amplification conditions were as follows: an initial denaturation step at 95 °C for 5 min, followed by 30 cycles of denaturation at 95 °C for 30 s, annealing at 55 °C for 30 s, and extension at 72 °C for 2 min, concluding with a final extension phase at 72 °C for 5 min. The PCR products were sent to Sangon Biotech (Shanghai) Co., Ltd. (Shanghai, China) for sequencing. The obtained sequences were then aligned with those in the NCBI database (https://blast.ncbi.nlm.nih.gov/Blast.cgi), and a phylogenetic tree for the R27 strain was constructed using MEGA7.0 software with the maximum likelihood method.

### 2.5. Removal Effect of R27 Strain on Cd^2+^

The R27 strain was inoculated into an LB liquid medium with concentrations of 0 mg/L and 2000 mg/L of Cd^2+^ and incubated at 37 °C with shaking at 200 rpm for a duration of 3 days. Samples were collected every 12 h and subsequently subjected to centrifugation and filtration using a 0.22 μm filter. The Cd^2+^ concentration in each filtrate was measured using an atomic absorption spectrophotometer (AA-680) according to the method of Shahid et al. [22]. The experiment was conducted in triplicate. Based on the detection results, the removal efficiency of Cd^2+^ by the R27 strain was calculated using the following formula:

$$R(\%)=\frac{C_{0}-C_{e}}{C_{0}}\times 100\%$$

where R is the Cd removal efficiency, C_0_ is the initial concentration of Cd (mg/L), and C_e_ is the remaining Cd concentration after culture (mg/L).

### 2.6. SEM, TEM, and EDX Analysis

SEM and TEM were performed to investigate the surface morphology and intracellular accumulation of Cd in the R27 strain under both non-Cd and Cd stress conditions. Following the cultivation of the R27 strain in an LB liquid medium with 0 and 2000 mg/L Cd^2+^ for 3 days, the cells were harvested and fixed in 2.5% glutaraldehyde for 12 h. This was followed by dehydration using ethanol and subsequent freeze-drying into powder. The samples were then coated with gold and mounted on a copper platform. Observations were performed using SEM (Nova Nano SEM 450, FEI, Hillsboro, OR, USA) [28] and TEM (Themis Z, Thermo Scientific, Munich, ND, USA) [29]. Additionally, energy-dispersive X-ray spectroscopy (EDX) (Nicolet is50, Thermo Fisher, Munich, ND, USA) equipped with SEM and TEM was utilized to analyze elemental composition [29].

### 2.7. FTIR Analysis

FTIR was employed to investigate the functional groups crucial for Cd absorption by the R27 cell surface. The R27 strain was cultured in an LB liquid medium with Cd concentrations of 0 mg/L and 2000 mg/L for a duration of 60 h at 37 °C, with stirring at 200 rpm. After centrifugation, R27 cells were harvested, vacuum-dried into a powder, and subsequently compressed into tablets at a 1:100 ratio for sample preparation. FTIR analysis was conducted using a Nicolet ls10 infrared spectrometer (ThermoFisher, Munich, ND, USA) according to the method of Iqbal et al. [30].

### 2.8. Pot Experiment

To investigate the influence of the R27 strain on the growth of lettuce (*Lactuca sativa* L.) under Cd stress, a pot experiment was conducted. Sandy loam soil samples were collected from Jiangxi Science and Technology Normal University (Nanchang, China). The initial concentration of Cd in the loam was measured at 0.12 mg/kg, with nitrogen at 548 mg/kg, phosphorus at 8.3 mg/kg, and extractable potassium at 412 mg/kg. CdCl_2_ was added to the soil to make 8 mg/kg of Cd^2+^. The soil was mixed, air-dried, ground, and autoclaved before the pot experiment. Lettuce seeds were subsequently planted in nutrient-rich soil and allowed to grow for 20 days to obtain seedlings with uniform size.

The R27 inoculum was prepared by cultivating the bacteria in an LB liquid medium. Following centrifugation at 5000× *g*, the cells were collected, washed three times with sterile water, and then diluted to a concentration of 10^8^ CFU/mL for the pot experiment.

Eighty lettuce seedlings, each with three leaves, were placed into pots (16.3 cm in width × 20 cm in depth) filled with 500 g of autoclaved soil. The soil was either untreated or treated with CdCl_2_, designated as CK and Cd treatment, respectively. Three days post-transplantation, half of the pots in both the CK and Cd treatments were inoculated with R27 cells in the rhizosphere of the lettuce seedlings, achieving a final concentration of 10^7^ CFU/pot. These inoculated pots were labeled as R27 and Cd + R27 treatment. The specific experimental design included the following: (1) CK (water only), (2) R27 (R27 inoculation alone), (3) Cd (Cd stress only), and (4) Cd + R27 (Cd stress combined with R27 inoculation), with each treatment comprising 20 pots. After 15 days of R27 inoculation, the shoot and root segments of the seedlings were collected for subsequent physiological measurements. The experiments were repeated three times as three biological replicates.

### 2.9. Chlorophyll Contents

The chlorophyll levels in the leaves were assessed using the procedure outlined by Ashraf and Iram [31], in which 0.25 g of the fresh leaf tissues was homogenized with 80% acetone and extracted overnight. Following extraction, the solutions were centrifuged at 12,000× *g* for 10 min, and the concentrations of chlorophyll a and b were determined by measuring the absorbance of the supernatant at wavelengths of 663 nm and 645 nm.

### 2.10. Measurement of Antioxidant Activities

The activities of catalase (CAT), peroxidase (POD), and superoxide dismutase (SOD) in the leaves were assessed according to the procedure outlined by Qiu et al. [32]. A total of 0.2 g of fresh shoot tissue from each sample was homogenized in a 50 mM phosphate buffer containing 1 mM EDTA. Following centrifugation, the supernatants were collected for the evaluation of enzymatic activities. Subsequently, the activities of CAT, POD, and SOD were measured by observing the reduction in H_2_O_2_ levels, guaiacol oxidation in the presence of hydrogen peroxide, and the ability to inhibit the photoreduction of nitro blue tetrazolium, respectively.

### 2.11. Transcript Analyses by qRT-PCR

The expression patterns of genes associated with Cd transport, including IRT1, *Nramp1*, *HMA2*, *HMA4*, *ZIP4*, and *ZIP12*, were examined in shoot and root tissues treated with R27 under both non-Cd and Cd stress conditions. Total RNA extraction from the tissues was performed using the Takara Mini BEST Plant RNA Extraction Kit (Takara, Chuo-ku, Osaka, Japan). Quantitative reverse transcription PCR (qRT-PCR) was conducted with SYBR Premix ExTaq (Takara, Chuo-ku, Osaka, Japan) employing gene-specific primers (Table S1). The experiment was replicated at least three times, and the expression levels of the genes were determined using the threshold cycle 2^−ΔΔCt^ method [33].

### 2.12. Measurement of Proline Content

The proline levels in the seedlings were determined using the procedure established by Bates et al. [34]. Initially, 100 mg of shoot tissue from each treatment group was homogenized in aqueous sulfosalicylic acid. The filtrate was then combined with acid-ninhydrin and glacial acetic acid and incubated at 100 °C for 1 h. Following this, the reaction mixtures were extracted with toluene, and the absorbance of the resulting mixture was measured at a wavelength of 520 nm.

### 2.13. Determination of Cd Contents in Plant Tissues

Following the overnight drying of the plant samples in an oven set to 105 °C, a total of 0.1 g of both shoot and root tissues was digested using HNO_3_-HClO_4_. The concentration of Cd was measured using a flame atomic absorption spectrometer. The root bio-concentration factor (BCF) and the translocation factor (TF) of Cd in the lettuce seedlings were calculated as follows [22]:

BCF = Root Cd concentration (μg/g)/Soil Cd concentration (μg/g)

TF = Shoot Cd concentration (μg/g)/Root Cd concentration (μg/g)

### 2.14. Statistical Analysis

All analyses were conducted using SPSS version 19.0. Significant differences in the data were evaluated through analysis of variance (ANOVA), followed by Duncan’s multiple range tests (*p* < 0.05) utilizing SPSS version 19.0 (SPSS Inc., Chicago, IL, USA). Additionally, a *t*-test was applied to assess statistical significance.

## 3. Results

### 3.1. Isolation of Cd-Resistant PGPR from Metal-Contaminated Soil

Six strains (R5, R12, R15, R27, R30, and R32) were isolated (tolerance to Cd concentrations higher than 500 µg/mL). The MIC and quantitative PGP traits, such as phosphate solubilization, siderophore production, and IAA production, of each strain were evaluated to identify Cd-resistant PGPR. The results revealed that the R27 strain exhibited the highest level of Cd tolerance, with an MIC of 2000 mg/L (Table 1), surpassing that of the other five strains. Furthermore, the R27 strain demonstrated all three assessed PGP traits, showing the highest phosphate solubilization capacity (385.11 mg/L) and IAA production (35.92 mg/L) (Table 1). In contrast, the remaining five strains displayed lower Cd tolerance (indicated by lower MIC values) and/or deficiencies in one or more PGP traits. Consequently, the R27 strain was ultimately selected as the Cd-resistant PGPR for subsequent experiments.

### 3.2. Identification of R27 Strain

**Figure 1:**
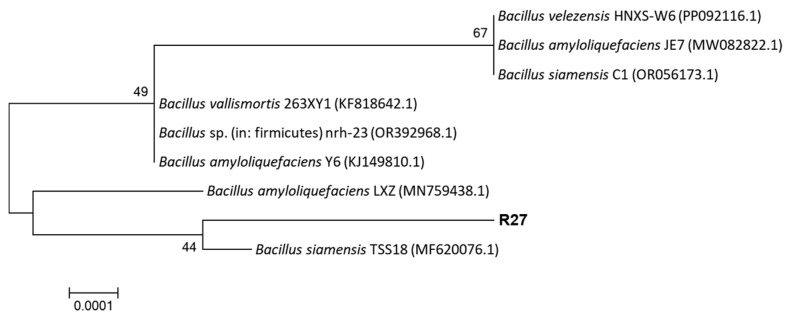
Phylogenetic relationships of 16S rRNA gene sequences of R27 strain.

Based on physiological and biochemical characteristics, the R27 strain was identified as Gram-positive bacteria. It exhibited positive results for starch hydrolysis, the gelatin hydrolysis test, indole reaction, oxidase reaction, hydrogen peroxidase reaction, V-P reaction, and nitrate utilization, and negative results for other indicators (Table 2). A 1454 bp 16S rRNA gene sequence of the R27 strain was obtained through PCR amplification, demonstrating 99.65% sequence identity with *Bacillus siamensis* TSS18 (MF620076.1). The phylogenetic tree indicated that the R27 strain and *B. siamensis* TSS18 clustered on the same branch (Figure 1). Therefore, based on physiological, biochemical, and molecular identifications, the R27 strain was classified as *Bacillus siamensis*.

### 3.3. Removal Effect of Cd^2+^ by R27 Strain

**Figure 2:**
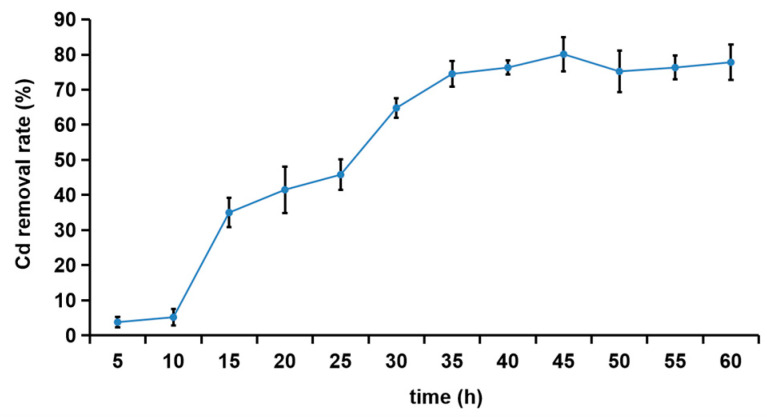
The removal effect of R27 on Cd^2+^ (*n* = 3, mean ± standard deviation).

As shown in Figure 2, the removal rate of Cd^2+^ by the R27 strain gradually increased from the 10th h to the 45th h, ultimately reaching a maximum of 80.1% at the 45th h. Following this peak, the removal rates began to stabilize. The relatively low removal rate of Cd^2+^ observed during the initial 10 h might be attributed to the R27 strain being in the lag and early logarithmic growth phases (Figure S1), during which the bacterial population remained relatively low, resulting in a correspondingly lower adsorption of Cd^2+^.

### 3.4. Adsorption Characteristic Analysis

**Figure 3:**
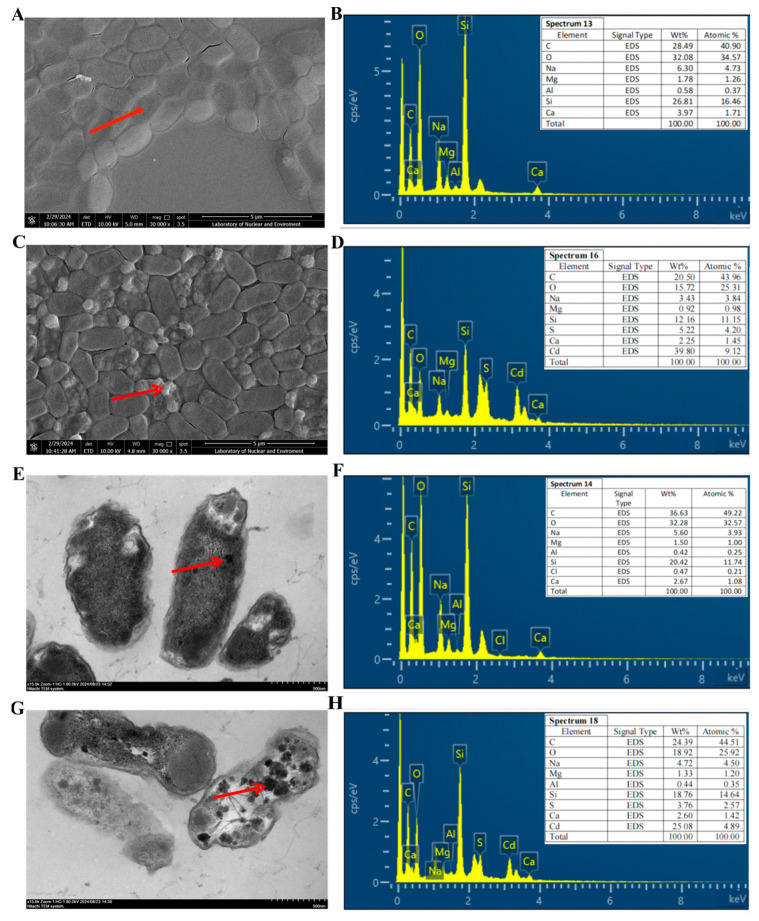
SEM and TEM images and EDX elemental analysis of R27 strain under non-Cd and Cd stress conditions. (**A**,**B**) SEM image and EDX spectrum of R27 under non-Cd stress condition; (**C**,**D**) SEM image and EDX spectrum of R27 under Cd stress condition; (**E**,**F**) TEM image and EDX spectrum of R27 under non-Cd stress condition; (**G**,**H**) TEM image and EDX spectrum of R27 under Cd stress condition. The red arrows indicated the marking selection point for spectrum analysis.

SEM and TEM were employed to examine the surface alterations and intracellular Cd accumulation of R27 bacterial cells under 0 mg/L and 2000 mg/L CdCl_2_ conditions (Figure 3). Although the size of the bacterial cells exhibited only minor differences between the two conditions, significant differences were observed in surface morphology. Specifically, the cells exposed to 0 mg/L CdCl_2_ displayed a smooth surface (Figure 3A), while those subjected to 2000 mg/L CdCl_2_ exhibited a rough surface characterized by irregular, large white crystalline particles (Figure 3C), which were hypothesized to be Cd deposits. EDX spectrum analysis confirmed the presence of Cd in the white regions of the bacterial cells in the 2000 mg/L CdCl_2_ condition, accounting for 9.12% of the total elemental composition (Figure 3B,D), thereby supporting this hypothesis. Moreover, the results of TEM depicted that Cd was also clearly accumulated within the cell, appearing as darker electron-dense particles (Figure 3E,G), with EDX results further corroborating these findings (Figure 3F,H). Collectively, these results indicate that R27 possessed the capability to sequester Cd through both extracellular and intracellular accumulation mechanisms.

### 3.5. FTIR Spectroscopy of R 27 Strain

**Figure 4:**
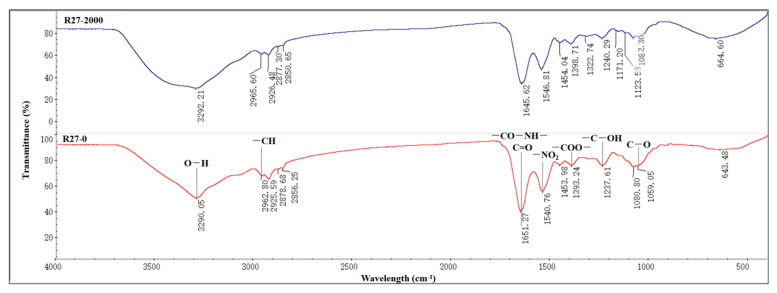
FTIR analysis diagram.

FTIR spectroscopy was conducted to further investigate the major functional groups present on the cell surface of the R27 strain that were involved in Cd absorption, and the results are shown in Figure 4. The FTIR spectrum of the R27 strain exhibited a broad peak at 3290 cm^−1^, attributed to O–H stretching vibrations. The O–H stretching occurred over a wide frequency range, indicating the presence of both free hydroxyl groups and bonded O–H bands associated with carboxylic acids. Additionally, a peak at 2962 cm^−1^ suggested the stretching vibration of –CH groups. The peak at 1651 cm^−1^ corresponded to the amide I band (–CO–NH–), associated with C=O stretching vibrations, while the absorption peak at 1540 cm^−1^ was linked to –NO₂ in the protein amide II band. Peaks observed at 1393 cm^−1^ were indicative of –COO– stretching vibrations. The peak around 1059 cm^−1^ was also attributed to the stretching vibration of C–O. Under Cd stress, the FTIR spectrum exhibited changes in the peak shape, position, and relative intensity of the characteristic peaks. Specifically, band shifting was observed, with the peak at 3290 cm^−1^ during the sorption process becoming stronger and broader, suggesting an active involvement of O–H in the complexation with Cd^2^⁺. Furthermore, a reduction in peak intensity at 1651 cm^−1^ and 1540 cm^−1^ was observed, potentially due to the interaction between Cd^2^⁺ and various O–H groups in the R27 strain, likely due to the coordination of C=O and –CO–NH– with Cd^2+^. Additionally, the transition of the C–O stretching vibration from 1059 to 1123 cm^−1^ distinctly indicated the interaction between Cd and the oxygen lone pair. These spectral shifts indicated that the O–H, C=O, –CO–NH–, and C–O groups played important roles in the adsorption of Cd.

### 3.6. Effect of R27 Strain on Growth of Lettuce Seedlings

**Figure 5:**
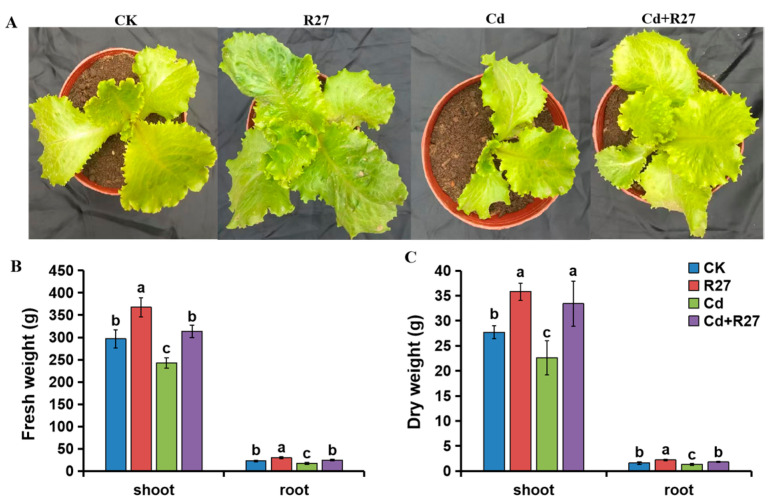
Effects of the R27 strain on the growth of lettuce seedlings under non-Cd and Cd stress conditions. (**A**) Lettuce seedling growth characteristics; (**B**) the fresh weights of lettuce; (**C**) the dry weights of lettuce. Blue, red, green, and purple bars represent CK (water only), R27 (R27 inoculation alone), Cd (Cd stress only), and Cd + R27 (Cd stress in combination with R27 inoculation) treatments, respectively. Different letters indicate statistically significant differences between the four treatments (Duncan’s multiple range tests, *p* < 0.05; *n* = 45, mean ± standard deviation).

The effects of the R27 strain on the growth of lettuce were assessed under both non-Cd and Cd stress conditions. The biomass of the lettuce seedlings inoculated with the R27 strain was obviously higher based on measurements of the fresh and dry weights of the plants (Figure 5). Compared with the non-inoculated seedlings, R27-inoculated seedlings exhibited 23.9% and 29.4% increases in shoot fresh weight in the non-Cd and Cd stress conditions, respectively. Additionally, differences in root biomass were also observed, with R27-inoculated seedlings demonstrating enhancements of 30.5% and 40.2% in root fresh weight under non-Cd and Cd conditions, respectively, compared to non-inoculated plants. Notably, growth-promoting effects were also evident in dry biomass. For the shoot parts, increases of 29.2% and 47.8% were detected in R27-inoculated seedlings under non-Cd and Cd stress conditions, respectively. Furthermore, the dry weights of root parts inoculated with R27 increased by 37.5% and 38.5%. These results revealed that the R27 strain could promote plant growth under both non-Cd and Cd stress conditions.

**Figure 6:**
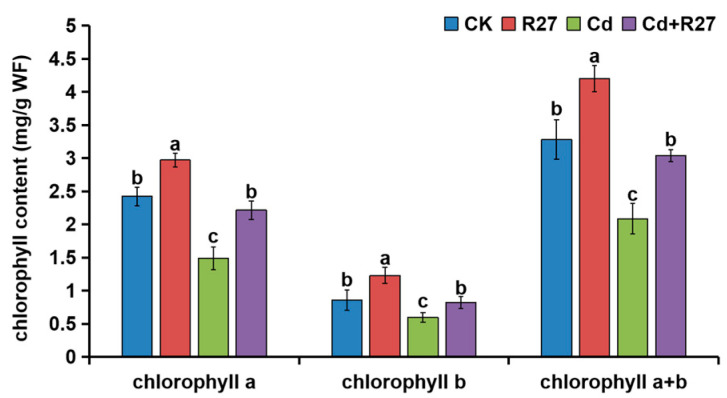
Effects of R27 on the chlorophyll contents of lettuce. Blue, red, green, and purple bars represent CK (water only), R27 (R27 inoculation alone), Cd (Cd stress only), and Cd + R27 (Cd stress in combination with R27 inoculation) treatments, respectively. Different letters indicate statistically significant differences between the four treatments (Duncan’s multiple range tests, *p* < 0.05; *n* = 6, mean ± standard deviation).

The contents of leaf chlorophyll a, b, and a + b in plants inoculated with R27 were also obviously higher under both non-Cd and Cd stress conditions (Figure 6). Under Cd stress, the leaf chlorophyll a, b, and a + b contents in lettuce seedlings inoculated with R27 increased by 49.0%, 37.7%, and 45.8%, respectively, compared with non-inoculated seedlings, which demonstrated that R27 could maintain leaf chlorophyll contents under Cd stress.

### 3.7. Effect of R27 Strain on Cd Accumulation, BCF, and TF

**Figure 7:**
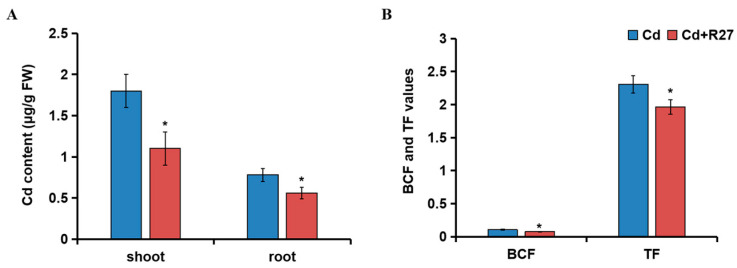
Effect of R27 strain on Cd content and Cd transport in lettuce. (**A**) The effects of R27 on the content of Cd; (**B**) the effects of R27 on the value of BCF and TF. Blue and red represent Cd (only Cd stress) and Cd + R27 (Cd stress + R27 inoculation) treatments. * Statistically significant differences (*p* < 0.05; *t*-test, *n* = 6, mean ± standard deviation).

The effects of R27 on the Cd content, BCF, and TF parameters are illustrated in Figure 7. Compared with Cd treatment alone, the Cd contents of the shoot and root treated with R27 significantly decreased by 38.9% and 28.2%, respectively (Figure 7A). Additionally, the BCF and TF values of Cd in R27-inoculated lettuce seedlings significantly reduced by 28.2% and 14.9%, respectively, compared to the non-inoculated plants (Figure 7B). These findings suggest that R27 had the potential to reduce the transport of Cd within lettuce, ultimately leading to reduced Cd accumulation in the plants.

### 3.8. Effect of the R27 Strain on the Expression of Genes

**Figure 8:**
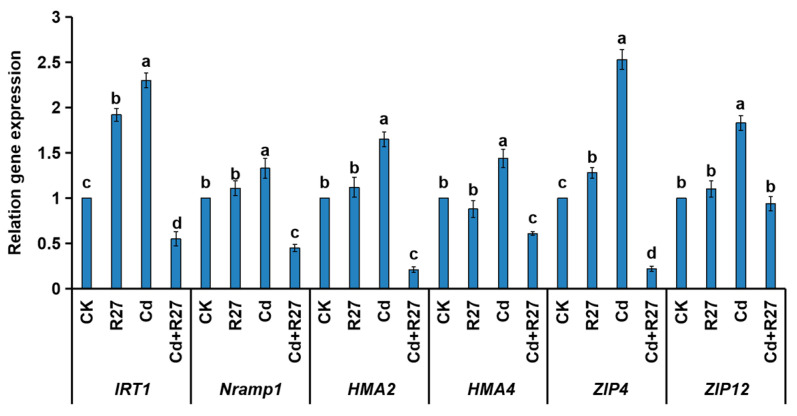
Effects of the R27 strain on the expression of genes related to Cd transport. Different letters indicate statistically significant differences between the four treatments (Duncan’s multiple range tests, *p* < 0.05; *n* = 3, mean ± standard deviation).

In order to clarify the mechanism by which R27 reduces Cd accumulation in lettuce seedlings, the expression of several genes responsible for Cd transports were detected by RT-PCR (Figure 8). Under Cd stress, the relative transcriptional levels of IRT1, *Nramp1*, *HMA2*, *HMA4*, *ZIP4*, and *ZIP12* were all significantly down-regulated by the R27 strain. These results indicate that R27 could effectively decrease the accumulation of Cd by reducing the transport of Cd in lettuce seedlings, which were consistent with the above results of lower BCF and TF values.

### 3.9. Effect of R27 on Antioxidant Activities and Proline Contents

**Figure 9:**
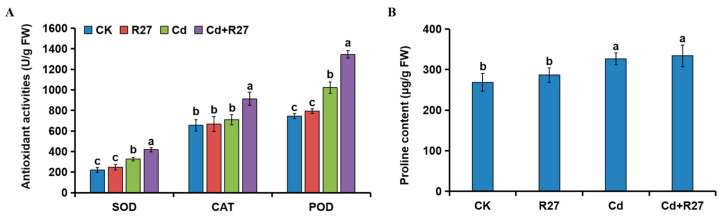
Effects of R27 on antioxidant activities and proline contents. (**A**) Effects of R27 on antioxidant activities of lettuce. Blue, red, green, and purple bars represent CK (only water), R27 (only R27 inoculation), Cd (only Cd stress), and Cd + R27 (Cd stress + R27 inoculation) treatments, respectively. (**B**) Effects of R27 on the contents of proline in plants. Different letters indicate statistically significant differences between the four treatments (Duncan’s multiple range tests, *p* < 0.05; *n* = 6, mean ± standard deviation).

Higher activities of the antioxidants SOD, CAT, and POD were observed in the seedlings inoculated with R27 (Figure 9). Under Cd stress, the activities of SOD, CAT, and POD in R27-inoculated lettuce significantly increased by 27.9%, 28.5%, and 31.7%, respectively, compared to the non-inoculated controls. Additionally, no differences in antioxidant activities were detected between the R27 and CK treatments.

Unexpectedly, the changes in proline contents in plants did not agree with those in antioxidant activities. No significant difference was observed in the proline contents of the plants treated with R27 and non-treated controls under Cd and non-Cd stress conditions.

## 4. Discussion

The application of PGPR is a widely used strategy for bioremediation aimed at alleviating heavy metal stress while simultaneously promoting plant growth [35,36]. The adsorption, transformation, and efflux functions of these microorganisms can immobilize, migrate, or transform heavy metal substances in the soil, thereby reducing the absorption of heavy metals by plants [37]. Numerous PGPR species have been identified that can sequester Cd and that exhibit significant tolerance to it, such as *Bacillus* sp. SDA-4 [22]; *Bacillus megaterium* NCT-2 and *Bacillus paranthracis* NT1 [38]; *Pseudomonas aeruginosa* KKU2500-8, KKU2500-9 [39]; *Salmonella enterica* 43C [40]; and *Enterobacter ludwigii* LY6 [41]. Previously, *Enterobacter* sp. strain EG16 [42] and *Bacillus* sp. strain SDA-4 [43] showed tolerance to Cd concentrations of 250 μg/mL and 3500 µg/mL, respectively. In the present study, among the six Cd-resistant isolated strains (R5, R12, R15, R27, R30, and R32), the R27 strain exhibited the highest level of Cd tolerance (2000 mg/L) (Table 1), which indicated a moderate level of Cd tolerance.

The R27 strain exhibited an obvious ability to adsorb Cd, achieving a considerable Cd^2+^ removal rate of 80.1% in liquid media (Figure 2), highlighting its significant potential in remediating Cd pollution. Different bacteria demonstrate differences in their capacities for and mechanisms of Cd^2+^ adsorption. Most studies indicate that functional groups such as hydroxyl, carboxyl, and amino groups on bacterial surfaces are crucial for binding with Cd^2+^ and other heavy metal ions. For instance, *Bacillus* sp. S3 predominantly utilizes the C=O, N–H, P=O, and O–H functional groups on its cellular surface to capture Cd^2+^. In contrast, the primary adsorption sites for Cd^2+^ on *Pseudomonas aeruginosa* JP-11 include –SH, N–H, O–H, C–O, and phosphoric acid. The FTIR analysis revealed that the R27 strain isolated in this study predominantly absorbed Cd^2+^ through the O–H, C=O, –CO–NH–, and C–O groups (Figure 4), significantly contributing to its enhanced Cd removal efficiency. Furthermore, TEM-EDX revealed that Cd was also detected within the R27 cells (Figure 3G,H). These results were consistent with previous studies demonstrating the intracellular accumulation of Cd in bacterial cells [42,43]. The dual mechanisms of extracellular and intracellular Cd accumulation in R27 may facilitate the sequestration of Cd in rhizospheric soil, thereby reducing Cd availability to plants and consequently limiting plant absorption of Cd.

PGPR possess the ability to absorb heavy metals from the environment and immobilize them in the soil, thereby reducing the uptake and accumulation of these metals by plants [43]. For instance, the strains Urease-producing *Enterobacter buccainensis* TJ6 and *Bacillus gigantium* HD8 effectively lowered the concentration of Cd by 75.3% to 85.8%, while simultaneously enhancing the biomass of lettuce by 31.3% to 55.2% [44]. Additionally, the introduction of the Cd-tolerant *Bacillus megaterium* L44 strain resulted in a 39.1% decrease in Cd concentrations in pepper fruits. Concurrently, significant improvements were observed in the root length, plant height, and fresh weight of the peppers, which increased by 65.7%, 20.6%, and 20.5%, respectively, compared to the control group (*p* < 0.05) [45]. Several other studies [46,47] have reported similar findings, demonstrating that PGPR not only enhances plant growth but also reduces Cd uptake, thereby enhancing the Cd resistance of plants. Similar results were observed in this study; R27 also promoted growth while reducing the absorption of Cd in lettuce. The lettuce seedlings treated with R27 exhibited significantly greater biomass and lower Cd concentrations compared to non-treated plants under Cd stress (Figure 5 and Figure 7A). To mitigate Cd toxicity, plants initiate various mechanisms that regulate the absorption, transport, and homeostasis of metals. This was confirmed by the result that R27 inoculation decreased Cd absorption (lower BCF), as evidenced by a lower BCF in lettuce, and restricted the translocation of Cd from the roots to the shoots, reflected in a lower TF (Figure 7B). Furthermore, these findings were corroborated by the down-regulation of six genes associated with Cd transport with R27 inoculation under Cd stress (Figure 8). These results corroborated with the findings of *Bacillus* sp. strain SDA-4, which was applied in Cd-stressed spinach (*Spinacia oleracea* L.) [22]. Additionally, the reduced Cd accumulation in lettuce might also be due to the Cd sequestration ability of R27, which sequestered Cd through both extracellular and intracellular accumulation pathways, as demonstrated by SEM, TEM, EDX, and FTIR analyses (Figure 3). Similar outcomes were observed in *Enterobacter* sp. S2, which exhibited the ability to bioaccumulate Cd and promote the growth of rice seedlings under Cd stress [43].

Chlorophyll plays a crucial role in the process of photosynthesis in plants, with its levels closely linked to the efficiency of these organisms in this process. Cd stress typically impairs photosynthetic activity by reducing chlorophyll levels within plants [13]. Numerous studies showed that PGPR could increase chlorophyll contents and promote plant growth [22,43,48]. For instance, *Bacillus siamensis* has been shown to elevate the concentrations of chlorophyll a, b, and total chlorophyll a + b in two varieties of wheat (*Triticum aestivum* L.) [49]. Similar results were observed in this study; the chlorophyll contents (a, b, and a + b) of the plants treated with *B. siamensis* R27 were significantly increased, by 49.0%, 37.7% and 45.8%, respectively, under Cd stress (Figure 6). The enhanced levels of chlorophyll are likely to alleviate the negative effects of Cd on photosynthesis, as evidenced by the significantly increased biomass of inoculated lettuce plants observed in this research.

Reactive oxygen species (ROS) serve as essential signaling molecules in plant development and their responses to abiotic stress [50]. However, excessive ROS resulting from heavy metal exposure can induce oxidative stress, adversely affecting plant growth [51], and oxidative stress in plants caused by Cd is widely recognized as a prevalent phenomenon [52]. In response to ROS-related stress, plants can activate both enzymatic processes (such as CAT, POD, and SOD) and non-enzymatic mechanisms (including proline and ascorbate) [53], and these mechanisms can be further enhanced by PGPR [43,54]. In this study, lettuce plants subjected to Cd stress exhibited increased activities of SOD, POD, and CAT, which were further augmented by R27 inoculation (Figure 9A). Rhizobacterial IAA could stimulate antioxidant enzymes and thus enhance plant tolerance to Cd stress [55]. Among the six isolated strains, R27 showed the highest capacity for IAA production (35.92 mg/L) (Table 1). Consequently, the increased activities of antioxidant enzymes in R27-inoculated plants could be attributed to its IAA synthesis. However, no significant increase in proline contents was detected in R27-inoculated plants under Cd stress (Figure 9B). Similar results were reported by [22]. It was possible that lettuce treated with R27 did not experience substantial Cd stress, resulting in lower proline levels in these seedlings.

## 5. Conclusions

In this study, the isolate strain R27, identified as *Bacillus siamese*, exhibited high resistance to Cd and possessed PGP traits. This strain demonstrated effective Cd adsorption capabilities that facilitated Cd sequestration. Moreover, the R27 strain helped lettuce plants tolerate Cd stress, with the underlying mechanisms likely involving increased antioxidant activities (POD, SOD, and CAT), reduced absorption of Cd by root parts (lower BCF), and inhibited transport of Cd to shoot parts (lower TF), as well as increased chlorophyll contents. These results showed that the R27 strain offers considerable potential for the bioremediation of Cd-polluted soils and ensures the safe production of vegetables in Cd-contaminated soil.

## Figures and Tables

**Table 1:** Characterization of the six isolates for MIC and plant growth-promoting traits.

Isolate	MIC (mg/L)	Phosphate Solubilization (mg/L)	IAA Synthesis (mg/L)	Siderophores (mg/L)
R5	500	257 ± 15.66	4.54 ± 0.28	8.37 ± 1.14
R12	1500	-	23.76 ± 1.76	-
R15	1000	7.73 ± 1.43	-	1.35 ± 0.56
R27	2000	385.11 ± 8.54	35.92 ± 2.15	3.34 ± 0.83
R30	1000	143.78 ± 5.69	18.92 ± 3.33	-
R32	1500	-	3.89 ± 0.98	6.44 ± 1.15

Note: Each value is the mean of three replicates (*n* = 3 ± standard deviation). “-” indicates no activity measured.

**Table 2:** Analysis of physiological and biochemical reaction characteristics of R27.

Physiological and Biochemical Experiments	Result
Gram’s stain test	+
Starch hydrolysis test	+
Gelatin hydrolysis test	+
Indole reaction	+
Methyl red test	−
Oxidase reaction	+
Hydrogen peroxidase reaction	+
V-P reaction	+
Nitrate utilization test	+
H_2_S production	−

Note: “+”: Positive effect, “−”: Negative effect.

## Data Availability

The 16 S rDNA sequence of R27 was submitted to NCBI, and the accession ID was 1144652. The R27 isolate was deposited at the China Center for Type Culture Collection (CCTCC) with designation number CCTCC NO: M 2023603.

## Supplementary Materials

The following supporting information can be downloaded at https://www.mdpi.com/article/10.3390/microorganisms12112321/s1, Figure S1: Growth curve comparison of R27 strain under Cd stress condition; Table S1: qRT-PCR primer sequences used in this study.
