# Supplementary material for: Cd-Resistant Plant Growth-Promoting Rhizobacteria Bacillus siamensis R27 Absorbed Cd and Reduced Cd Accumulation in Lettuce (Lactuca sativa L.)
Source: Microorganisms. 2024 Nov 15;12(11):2321. doi: 10.3390/microorganisms12112321 (PMC11596447; doi:10.3390/microorganisms12112321)
Supplement: Supplementary file 1 [file microorganisms-12-02321-s001.zip › microorganisms-3295967-supplementary.pdf]

Supplemental Data

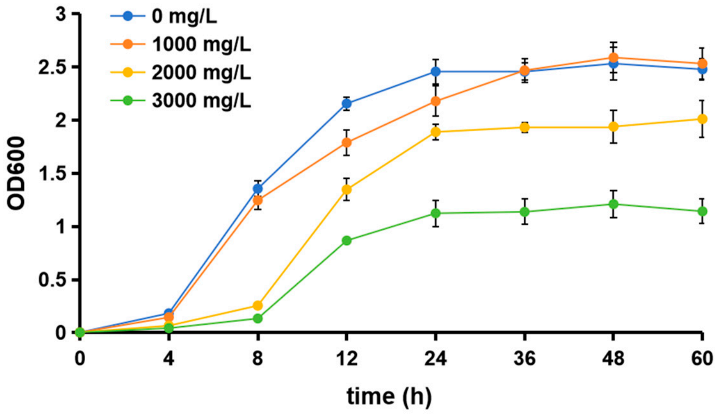

Figure S1. **Growth curve comparison of R27 strain under Cd stress conditon.**

Table S1. qRT-PCR primer sequences used in this study.

Gene	Forward primer	Reverse Primer
IRT1	CAACCCGATCCCAAGAGCAA	GGCGGAGTACAAGCCGTTAA
Nramp1	TGGAAAGGCGGTAGCAGAGC	TGCAATAGCGGTCCCAGCAC
HMA2	TCGGAGGTGCCTTTGATTGA	CAGGACGGGGTCGTGAAGTA
HMA4	TCTCAATCCGCCGCTAATCA	TCATCCCGTCTCCCACCATA
ZIP4	ACATGGCGTTGGTCGATCTTCTTG	GACGGCGGCGAAAGATTTGATTTG
ZIP12	CCTGCTCTCCACCTTCACAACAC	GCAGCAATGGCGATGACTTTAAGC
Actin	GTGAGTGAAGAAGGGCAATG	CACTTTCAACCCGATTCACC
